# Cardiovascular Imaging Applications, Implementations, and Challenges Using Novel Magnetic Particle Imaging

**DOI:** 10.3390/bioengineering12111235

**Published:** 2025-11-11

**Authors:** Muhiddin Dervis, Ahmed Marey, Shiva Toumaj, Ruaa Mustafa Qafesha, Doaa Mashaly, Ahmed Afify, Anna Langham, Sachin Jambawalikar, Muhammad Umair

**Affiliations:** 1 Faculty of Medicine, Harvard Medical School, Massachusetts General Hospital, Boston, MA 02114, USA; 2 Faculty of Medicine, Alexandria University, Alexandria 21521, Egypt; 3 Faculty of Medicine, Urmia University of Medical Sciences, Urmia 51369-67765, Iran; 4 Department of Anatomy and Embryology, Faculty of Medicine, Al-Quds University, Jerusalem P.O. Box 89, Palestine; 5 Faculty of Medicine, October 6 University, Giza 12566, Egypt; doaamashaly@gmail.com; 6 Faculty of Medicine, Benha University, Benha 13518, Egypt; 7 Department of Materials Science and Engineering, Johns Hopkins University, Baltimore, MD 21218, USA; 8 Department of Radiology, Columbia University, New York, NY 10027, USA; 9 Department of Radiology, Columbia University Irving Medical Center, New York, NY 10027, USA; 10 Department of Radiology, Johns Hopkins University, Baltimore, MD 21218, USA

**Keywords:** Magnetic Particle Imaging (MPI), superparamagnetic iron oxide nanoparticles (SPIONs), cardiac imaging, Field-Free Point (FFP), Field-Free Line (FFL)

## Abstract

Magnetic Particle Imaging (MPI) is a new type of tracer-based imaging that has great spatial and temporal resolution, does not require ionizing radiation, and can see deep into tissues by directly measuring the nonlinear magnetization response of superparamagnetic iron oxide nanoparticles (SPIONs). Unlike Magnetic Resonance Imaging (MRI) or Computed Tomography (CT), MPI has very high contrast and quantitative accuracy, which makes it perfect for use in dynamic cardiovascular applications. This study presents a full picture of the most recent changes in cardiac MPI, such as the physics behind Field-Free Point (FFP) and Field-Free Line (FFL) encoding, new ideas for tracer design, and important steps in the evolution of scanner hardware. We discuss the clinical relevance of cardiac MPI in visualizing myocardial perfusion, quantifying blood flow, and guiding real-time interventions. A hybrid imaging workflow, which improves anatomical detail and functional assessment, is utilized to explore the integration of MPI with complementary modalities, particularly MRI. By consolidating recent preclinical breakthroughs and highlighting the roadmap toward human-scale implementation, this article underscores the transformative potential of MPI in cardiac diagnostics and image-guided therapy.

## 1. Introduction

Magnetic Particle Imaging (MPI) is a new modality of tomographic imaging that employs the nonlinear magnetic properties of superparamagnetic iron oxide nanoparticles (SPIONs) to provide high-resolution, high-sensitivity images. MPI lacks artifacts caused by air or metal compared to Computed Tomography (CT) imaging, provides ionizing radiation-free imaging, and has very high tracer sensitivity with linear quantification capabilities. Because of these benefits, MPI is gaining popularity to investigate its potential future diagnostic and interventional uses. MPI uses SPIONs as tracers in a magnetic field that changes over time. When used as a contrast agent in Magnetic Resonance Imaging (MRI), SPIONs change the signals of existing tissue to create contrast. In MPI, however, SPIONs instead work as tracers as the only source of signal generation, making functional imaging possible [1].

Combining anatomical information from SPIONs as MRI contrast agents with functional information as MPI tracers enables MPI-MRI fusion imaging, which could potentially serve as a radiation-free modality of functional imaging with anatomical superimposition using a single agent-SPION. Moreover, its high temporal resolution enables real-time navigation and interventional applications. The Food and Drug Administration (FDA) has approved certain SPIONs as contrast agents, and they are safe for individuals with Chronic Kidney Disease (CKD) because they are primarily cleared via the hepatobiliary pathway rather than the kidneys. After intravenous administration, SPIONs are predominantly sequestered by the Mononuclear Phagocyte System (MPS), particularly Kupffer cells in the liver and macrophages in the spleen, where they undergo enzymatic degradation into bioavailable iron ions. These ions are subsequently integrated into physiological iron metabolism pathways, including ferritin and hemosiderin storage and hemoglobin synthesis, thereby minimizing renal burden [2,3].

MPI utilizes magnetic field gradients to create “Field Free Points (FFPs)” or “Field-Free Lines (FFLs)” where magnetic nanoparticles interact with an AC excitation field [4,5]. The AC excitation causes the nanoparticles’ magnetization to oscillate nonlinearly, generating harmonics that can be detected and used to reconstruct high-resolution images [6]. MPI provides several distinct advantages, including rapid image acquisition, deep tissue penetration, high sensitivity, high temporal resolution, and a non-invasive nature with zero radiation exposure [7,8]. Considering these advantages, MPI demonstrates significant potential for medical applications, including angiography, cancer diagnosis, cell tracking, and cardiac imaging and interventions [9,10].

MPI has demonstrated potential in cardiac imaging for visualizing the beating heart, assessing myocardial perfusion and viability, and obtaining detailed images of cardiovascular structures at the preclinical stage, primarily in small animal models such as mice and rats, as no human studies have been reported to date [5,11]. Nonetheless, the rapid temporal resolution of MPI, precise cardiac imaging, confronts challenges akin to those in MRI, including motion artifacts induced by cardiac and respiratory cycles. For synchronizing changes in tracer signals with the cardiac cycle, ECG gating and breath-hold or respiratory-gated acquisitions are essential. The information obtained from retrospective cardiac MRI gating methods may lead to the creation of novel MPI protocols that allow for better temporal alignment and quantitative evaluation of myocardial perfusion and ventricular dynamics [12].

Early ultrafast MRI studies, such as first-pass cardiac perfusion imaging, illustrated the importance of detecting rapid hemodynamic alterations during contrast passage. Likewise, MPI’s millisecond temporal resolution enables the visualization of first-pass tracer kinetics and beat-to-beat perfusion patterns without ionizing radiation, potentially addressing certain limitations of MR perfusion techniques [13]. MPI has additionally demonstrated a lot of potential in pulmonary imaging for accurately finding perfusion defects, diagnosing conditions like pulmonary embolism, and measuring pulmonary vascular permeability [14,15]. Vascular imaging has also made a lot of progress due to MPI’s high-resolution capabilities, especially when it comes to measuring vascular stenosis and visualizing blood flow. Studies have indicated that MPI can accurately measure the structure of blood vessels and detect pathological changes [16,17,18,19].

MPI has a lot of potential in cardiovascular and interventional radiology. Digital Subtraction Angiography (DSA) and X-ray fluoroscopy are two common interventional radiology methods that only provide images in two dimensions. They use ionizing radiation and iodine-based contrast agents. Conversely, MPI may offer a safer, higher-resolution, real-time imaging alternative devoid of ionizing radiation exposure, contingent upon validation in upcoming clinical studies [20]. Researchers have examined various techniques for visualizing interventional instruments through MPI, including the application of SPIONs for labeling instruments, thereby enhancing clarity and accuracy in imaging during procedures [21,22]. Multi-color MPI techniques have also greatly improved their performance by enabling the differentiation of signals from diverse particle types or environments. This is critical for procedures that need to accurately locate and navigate multiple devices [23]. These multi-contrast approaches have further enhanced MPI efficiency by allowing simultaneous visualization of different tracers or materials within a single imaging field, thereby improving real-time vascular and interventional imaging through better differentiation between SPION-labeled devices and surrounding tissues [24].

Recent evaluations have indicated advancements in MPI technology, particularly in tracer design, multimodal applications, and oncology-related imaging [7,25]. However, there is still a lot of research to be conducted on using these devices for cardiovascular applications and combining them with MRI for dynamic hemodynamic assessment. This review analyzes advancements in preclinical cardiac and vascular MPI. We concentrate on the advancement of tracers, hybrid MPI-MRI systems, and interventional applications, while addressing physiological imaging challenges including cardiac gating, perfusion quantification, and hardware scalability. This work offers a thorough analysis of the potential of MPI to enhance cardiovascular diagnostics and interventions by highlighting translational barriers and future directions.

## 2. History and Development of MPI

MPI is a relatively new imaging modality that has rapidly progressed from a conceptual discovery to a promising tool for biomedical and cardiovascular applications. Its unique ability to provide quantitative, radiation-free, and high-temporal-resolution imaging has driven growing interest in both preclinical and translational research. This section outlines the historical milestones and key technological developments that have shaped the evolution of MPI into a potential next-generation technique for cardiovascular imaging.

### 2.1. History of MPI

Bernhard Gleich created MPI at Philips Research Laboratories in Hamburg; the idea was patented in 2001, and the MPI scanner’s proof of concept was published in 2005. Initial phantom studies revealed several technical challenges: long acquisition times, limited to static imaging, mechanical constraints, and required tracer doses above clinically approved levels for the MRI contrast agent [26].

**Figure 1:**
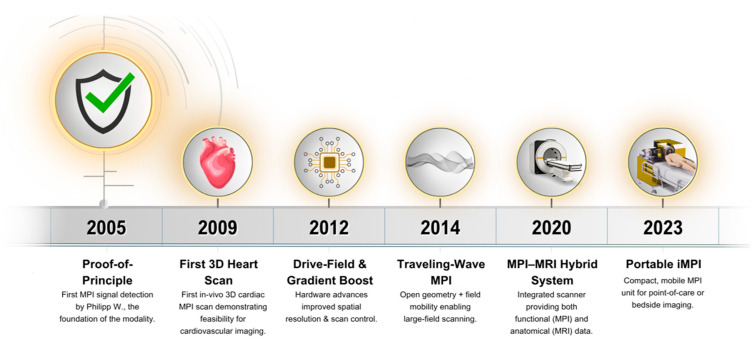
Timeline of Cardiac MPI Hardware Milestones (2005–2023).

Subsequent hardware iterations added extra coil pairs, reducing acquisition times to 4 ms and enabling real-time imaging at 25 frames s^−1^, although tracer concentrations were still too high for patient use [27]. In 2009, the first three-dimensional in vivo dataset captured a mouse’s beating heart at 46 frames s^−1^ with clinically acceptable tracer doses, aided by a wideband low noise amplifier [5]. A second-generation 12 cm bore scanner followed in 2010, expanding its field of view to human dimensions; however, this equipment required drive-field strengths that would cause tissue-level heating [28]. The 2011 focus-field concept enlarged the Field of View (FOV) to 50 × 43 × 28 mm^3^, proving volumetric imaging of several organs [29]. Figure 1 presents a timeline of key milestones in cardiac MPI hardware development, from the initial proof-of-principle to the latest portable iMPI systems. Commercial preclinical scanners are now available, fueling application-driven research, including cardiovascular imaging, even though routine human use awaits regulatory approval [30].

### 2.2. Fundamental Principles and Mechanics

By taking advantage of SPIONs’ nonlinear magnetization response, which is characterized by the Langevin function, to oscillating driving fields and static selection field gradients, MPI maps the geographic distribution of SPIONs [4,27,31,32,33].

In addition to the Langevin function, which assumes instantaneous alignment of magnetic moments with the applied field, other mathematical formulations have been proposed to capture more realistic magnetization dynamics in superparamagnetic and ferromagnetic nanoparticles. The Landau–Lifshitz–Gilbert (LLG) equation describes the precessional motion and damping of magnetic moments under the influence of effective magnetic fields, accounting for both torque and energy dissipation during magnetization processes. This formulation is especially beneficial for simulating the time-dependent response of nanoparticles to alternating magnetic fields in MPI [34].

The Fokker–Planck equation provides a statistical framework for delineating the stochastic thermal fluctuations and Brownian rotational diffusion of magnetic moments, thereby connecting microscopic thermal noise with macroscopic magnetization behavior [35,36]. Moreover, the modified Jiles–Atherton (J–A) model enhances classical hysteresis theory by integrating parameters that define domain wall pinning, magnetization reversibility, and energy dissipation mechanisms. This model makes it possible to more effectively demonstrate nonlinear and hysteretic magnetization loops that happen in ferromagnetic materials. It additionally helps to clarify the energy dissipation and relaxation processes that affect the generation and resolution of MPI signals [37].

**Figure 2:**
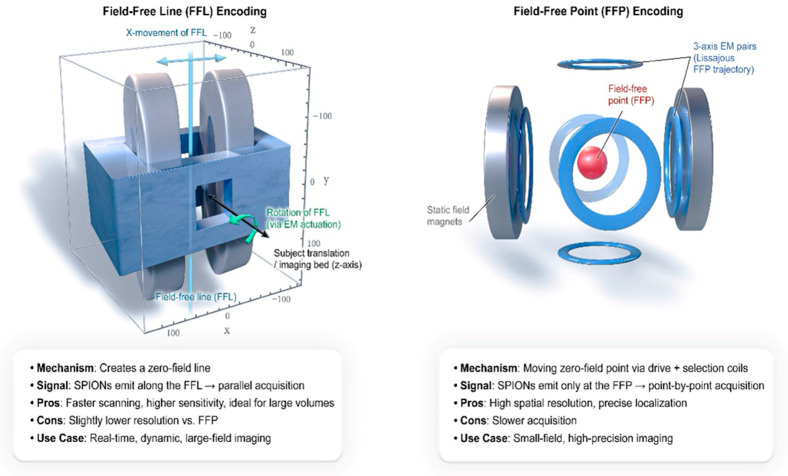
MPI Physics Primer: Field-Free Point vs. Field-Free Line Encoding.

To make high-resolution images, frequency domain filtering is used to detect and separate the higher-order harmonics, primarily the odd harmonics of the fundamental drive frequency, which the SPIONS generate at the FFP or FFL. Recent FFL encoding schemes boost sensitivity and enable computationally efficient Radon transform reconstruction [38]. The FFP is moved across the volume of interest either mechanically or magnetically to accomplish spatial encoding [24,25]. Figure 2 illustrates the difference between FFP and FFL encoding strategies. It demonstrates how various types of scanners affect acquisition speed, resolution, and use cases.

MPI’s high sensitivity and strict linearity between signal and tracer mass come from eliminating Barkhausen jumps and analyzing drive-field harmonics [39].

### 2.3. Brownian vs. Néel Relaxation

The behavior of SPION magnetization is consistent with Langevin statistics; magnetic relaxation may be attributed to either internal magnetic moment flipping (Néel relaxation) or physical particle rotation (Brownian relaxation) [40,41,42]. Brownian relaxation processes dominate at low frequencies and are temperature dependent, while Néel relaxation governs higher frequency behavior. Understanding both is essential for designing tracers and pulse sequences that maximize the signal in fast-moving organs such as the heart [43].

### 2.4. Tracers—SPIONs

MPI currently relies on SPION tracers, whose magnetization can be detected directly and quantified. Resovist^®^ (ferucarbotran), hydrodynamic diameter ~60 nm, yields a strong signal, although only ~3% of its iron core contributes effectively [31,44]. Real-time cardiac MPI in mice has been demonstrated at 20 nmol Fe L^−1^, achieving up to 50 × 3D volumes s^−1^ [5]. Ongoing nanoparticle engineering aims to extend blood half-life, enhance relaxivity, and enable multicolor imaging for simultaneous perfusion and device tracking [27,31,45].

### 2.5. Comparison with Other Imaging Modalities

MPI offers unique advantages compared to other imaging modalities, though it relies on the distribution of magnetic nanoparticles and requires the injection of a tracer, without providing morphological/anatomical information. Important imaging features of MPI include sensitivity, quantifiability, spatial resolution, and temporal resolution, much like the majority of medical imaging [46].

Unlike imaging methods like CT and MRI, MPI directly identifies SPIONs rather than depending on changes in tissue contrast [43]. In contrast to PET and SPECT, which also use tracers, MPI is a radiation-free technique, making it safer for repeated use [46]. Additionally, while MRI detects signals from hydrogen protons and provides structural images, MPI detects iron oxide nanoparticles directly, offering superior sensitivity for tracer detection, and imaging of distribution over time, making it a modality of choice for biodistribution-based imaging, for example [47,48].

A key advantage of MPI is its ability to provide a direct correlation between image signal and material concentration, enabling real-time imaging with high quantitative accuracy [43]. Unlike PET and SPECT, which suffer from signal attenuation due to photon scattering or absorption, MPI maintains a high signal-to-noise ratio and does not experience background interference from biological tissues [47]. In clinical applications, MPI can be complemented by anatomical imaging methods like MRI or CT to provide both structural and functional insights [48]. A quantitative comparison of MPI with other major imaging modalities is summarized in Table 1.

## 3. Potential Applications of MPI in Cardiac Imaging

Over the past decade, MPI has demonstrated strong potential in preclinical cardiovascular research, particularly for the real-time visualization of cardiac motion and perfusion. Early in vivo studies demonstrated the feasibility of capturing the dynamics of a beating mouse heart with high temporal and spatial resolution, while later investigations focused on improving tracer performance and image quality. Table 2 summarizes representative studies that have evaluated MPI for cardiac imaging, outlining their experimental designs, tracer types, and major findings relevant to future clinical translation.

Weizenecker et al. [5] were the first to explore the potential of MPI for high-resolution, real-time cardiovascular imaging using a clinically approved MRI contrast agent, Resovist, in live mouse models. The research aimed to validate MPI’s efficacy and sensitivity in capturing detailed anatomical features of a beating heart at safe dosages for human applications. The experiments involved in vivo scans on 18 mice with varying Resovist concentrations, correlating MPI data with anatomical landmarks from post-scan MRI images to assess spatial and temporal resolution. The findings demonstrated that MPI could successfully visualize cardiovascular structures with high precision and stability, suggesting its promise as a viable clinical imaging technique for detailed, real-time cardiovascular assessments [5].

The first MPI scanner had a small FOV; thus, many approaches and hardware advancements were made to fix this problem. In 2014, a new type of scanner called Traveling Wave Magnetic Particle Imaging (TWMPI) became available [50]. Vogel et al. examined the TWMPI scanner in 2016 to see if it could identify a beating mouse heart [11]. Using a dynamic Linear Gradient Array (dLGA), TWMPI makes it possible to scan a whole volume the size of a mouse by creating two FFPs with opposite gradient slopes [51]. Each coil has sinusoidal currents and phase shifts that make a moving magnetic field along the main axis. This lets you encode line by line, called Line Scanning Mode (LSM). The downside to this technique is lower radial resolution [50]. To solve this problem, the Simultaneous Scanning Method (SSM) was created [52,53].

SSM speeds up measurements by scanning a whole 2D slice at once and improves in-plane spatial resolution. Stacking several parallel slices can make a full 3D volume, but SSM made the resolution worse along the third dimension, making “quasi”-projections instead of true 3D images. The SSM for TWMPI is great for quick tasks like taking real-time measurements in small rodents because it rapidly accumulates 2D data over a large FOV, covering an entire mouse-sized sample. Vogel et al. showed that in vivo imaging is possible with a TWMPI scanner that has a high enough temporal resolution to see how a mouse heart beats [11].

Multimodal imaging techniques are very important for clinical use and diagnosis. When MPI is used with other imaging methods like CT or MRI, anatomical information can be superimposed on the functional information obtained using MPI [54]. Weizenecker et al. were the first to use MPI and MRI together for morphological reference in 2009 [5]. Since then, more multimodal MPI applications have been released [47,55,56]. However, all of these multimodal studies used two different imaging equipment, which made the workflow very difficult and made it hard to handle the animals. Fiducial markers were needed to make it easier to register multimodal images with the right level of spatio-temporal fidelity. As a result, numerous hybrid MPI scanner designs were made to lower the inaccuracies that occur when objects are moved or repositioned between different modalities [57,58].

Franke et al. [57] were the first to employ a hybrid multimodal system to check the heart in situ. They used a pre-clinical MPI-MRI hybrid imaging system that had a full coil setup that let them encode space, excite signals, and receive signals for both imaging modes without having to move the animals around. They used MRI to obtain three-dimensional anatomical information and MPI bolus tracking to monitor superparamagnetic iron oxide nanoparticles. The hybrid approach made it easier to arrange MR-based MPI FOV, analyze the anatomy of the heart in cross-section, accurately co-register dual-modal information, and perform MPI-based hemodynamic functional analysis [57].

The morphological data from the MRI mode were enough to manually separate the four heart chambers and the lungs. They used this technique in real-time to track tracer distribution over time and to derive velocity estimates across major cardiac vessels, including the ascending aorta and pulmonary artery, within a retrospectively gated virtual cardiac cycle. These measurements primarily reflected the bulk flow velocities in the great vessels and intracardiac chambers but were not optimized for small-scale flow across cardiac valves.

**Figure 3:**
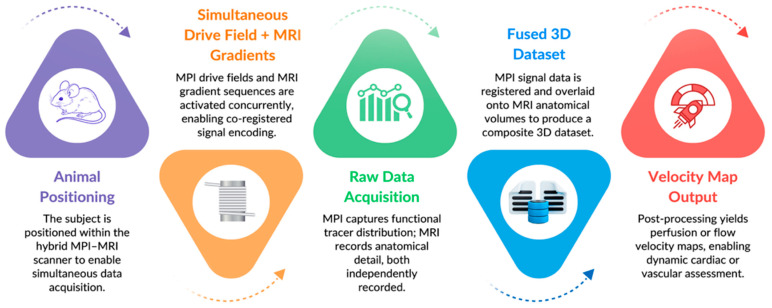
Hybrid MPI-MRI Workflow Diagram.

Additionally, it was possible to estimate derived parameters such as cardiac output fractions and pressure gradients based on these large-vessel velocity measurements. The MPI-MRI hybrid imaging system, therefore, enabled the rapid, noninvasive, and quantitative assessment of central hemodynamic parameters in situ, which may have future applications in ICU patients’ post-coronary artery bypass surgery, during cardiogenic shock, or for early stage cardiovascular disease monitoring [57]. The integrated acquisition and co-registration process of the hybrid MPI-MRI pipeline is visualized in Figure 3, demonstrating a streamlined workflow from animal setup to fused velocity map output.

**Figure 4:**
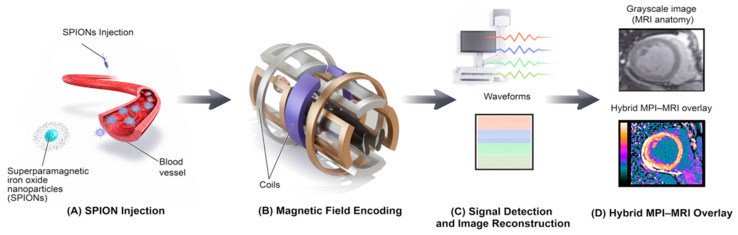
Schematic Workflow of MPI, from Tracer Injection to Hybrid Image Formation.

To provide a clearer conceptual overview of how the imaging pipeline operates, Figure 4 illustrates the complete schematic workflow of MPI from tracer injection to hybrid image formation. It highlights each step of the process, beginning with SPION administration, magnetic field encoding, and signal reconstruction, and concluding with a hybrid MPI-MRI overlay for anatomical correlation. This schematic complements the experimental workflow shown in Figure 3 by summarizing the technical sequence common to both preclinical and hybrid MPI-MRI applications.

- (A) SPION Injection:
  SPIONs are injected intravenously and circulate within the bloodstream.
- (B) Field Encoding:
  Within the MPI scanner, oscillating magnetic fields generated by gradient coils spatially encode the nanoparticles’ magnetic responses.
- (C) Image Reconstruction:
  The resulting voltage signals are detected and computationally reconstructed into a spatial image representing tracer distribution.
- (D) Hybrid Overlay:
  The reconstructed MPI image is co-registered and overlaid with structural MRI data to create a hybrid image that combines functional tracer localization with anatomical context.

These studies demonstrate the great future potential for MPI as a clinical imaging technique for cardiovascular assessments due to its high-resolution, real-time imaging capabilities, successful integration with other modalities like MRI, and innovative scanner designs such as TWMPI that improve the field-of-view and spatial resolution.

## 4. Evolution and Improvement of MPI Tracers and Hardware Systems

The continuous evolution of MPI technology has been driven by advancements in both tracer materials and hardware design. Early studies focused on improving the biostability, circulation half-life, and magnetic responsiveness of SPION tracers to enhance imaging performance in cardiovascular applications. Concurrently, hardware innovations, such as specialized receiver coils and human-scale scanner prototypes, have significantly increased MPI’s sensitivity, spatial resolution, and translational potential. Table 3 summarizes representative studies highlighting key developments in tracer optimization and hardware improvement that have collectively advanced MPI toward clinical readiness.

MPI is an exciting new area of three-dimensional imaging that takes advantage of two important properties of SPIONs: their nonlinear magnetization response and their ability to become magnetically saturated in strong magnetic fields. This lets MPI provide very high temporal and spatial resolution along with high signal-to-noise ratios [61,63]. For MPI to function effectively, it depends on two critical components: specialized MPI scanners and the right tracer materials. The most efficient tracers are long-circulating SPIONs that can send a strong signal through the bloodstream after just one injection. This makes it simple to conduct both diagnostic scans and subsequent interventions seamlessly.

Resovist was one of the earliest tracers among the SPIONs to be tested for MPI and was the first to be approved as a contrast agent for MRI [44]. However, it is noteworthy how Resovist works in the context of MPI, especially when it comes to its blood half-life. The reticuloendothelial system removes larger Resovist particles from the bloodstream faster than smaller ones, so not all of them add the same amount to the MPI signal. Haegele et al. [22] investigated this by precisely measuring the blood half-life of Resovist when used in MPI. The study employed MPS to enhance the existing knowledge derived from the MRI literature regarding the distribution of this tracer. They determined that Resovist demonstrates a rapid decline in blood MPI signal, rendering it unsuitable for application as a long-circulating tracer. Nonetheless, it was established that Resovist can be effectively employed as a bolus tracer in cardiovascular MPI applications, including early phase dynamic angiography imaging [22].

One of the most important things that can change the blood half-life of SPION tracers is the coating on the tracers. Surface coatings are crucial for MPI tracers, maintaining their essential properties for optimal performance in physiological settings. These coatings protect organisms by keeping the hydrophobic SPION cores from coming into contact with the environment. The design of the MPI tracers affects their blood half-life and distribution. Consequently, modifying SPIONs with different coatings can lead to altered tracer performance, and their structure determines the biodistribution of MPI tracers [48,64].

The size and size distribution of SPION tracers, in addition to their surface coatings, are important factors that determine their magnetic behavior and physiological performance [25]. The magnetic core diameter directly influences saturation magnetization and relaxation dynamics (Néel and Brownian), which together define MPI signal strength and spatial resolution. Both theoretical modeling and experimental studies have indicated that iron-oxide cores within the range of ~20–30 nm exhibit optimal magnetization behavior and signal response. This range balances superparamagnetic stability with efficient magnetization reversal and minimal aggregation in biological media [7,65,66,67]. In contrast, tracers with hydrodynamic diameters above roughly 50 nm, for example, Resovist with a mean size near 60 nm, are rapidly cleared by the mononuclear phagocyte system, reducing circulation time and limiting vascular imaging efficiency [59,68]. These findings highlight that MPI tracer performance is critically dependent on both core and hydrodynamic size, as smaller cores reduce magnetic moments and hydrodynamic properties influence in vivo behavior, including circulation and biocompatibility. Careful design of both core and surface properties is therefore essential to achieve optimal tracer performance in physiological settings [30].

Khandhar et al. [60] refined the surface coatings of SPIONs with polyethylene glycol (PEG) to enhance their in vivo and in vitro performance, resulting in improved colloidal stability and prevention of SPION aggregation. This modification led to a longer blood half-life for SPIONs while preserving their signal response. They further compared the in vivo circulation time of SPIONs based on their hydrodynamic diameter and discovered that clustered SPIONs stayed in the blood for five times less time than individually coated SPIONs [60].

Therefore, making MPI SPION tracers with longer blood half-lives requires a lot of thought, since this might greatly improve vascular imaging and make molecular targeting easier. These kinds of modifications via coating are very important for the specific imaging application of MPI. Such advancements are critical for delineating tumor vascularity, for example, using MPI, as the effectiveness of nanoparticles in accumulating within tumors depends on factors including biodistribution and blood-pool and tissue-level pharmacokinetics, their hydrodynamic size, surface coating, and surface charge [64]. For example, highly metastatic breast cancer was observed using MPI by labeling the monocytes of the liver and spleen with ferucarbotran and ferumoxytol, which infiltrated the tumor [69].

To explain more about why SPION tracers with longer blood half-lives are needed, Kaul et al. compared LS-008, a novel monodisperse and size-optimized MPI tracer, to Resovist in 2017 [49]. The study found that LS-008 had a longer blood half-life of 88 min, compared to Resovist’s 13 min. This made late-phase imaging viable, making it possible to visualize the perfusion pattern through certain organs, especially the liver and kidneys, even after the first pass kinetics. In in vitro experiments, LS-008 demonstrated a threefold increase in signal strength compared to Resovist. They also compared LS-008 and Resovist to study organ-level perfusion and SPION distribution. They found that LS-008 made the liver and kidneys easier to visualize, kept the signal levels stable in these organs over time, and had a slower signal decay in blood than Resovist, which showed a rapid signal shift from blood to liver.

In addition to LS-008, the FDA approved Ferumoxytol, an iron oxide nanoparticle that can be administered intravenously, to treat anemia in people with chronic renal disease. It has notable physical, biological, and chemical features, like super-paramagnetism, biocatalytic activity, and immunomodulatory behavior, which can make it valuable for many biomedical applications. It works as an iron supplement, improves MRI images, and makes reactive oxygen species when combined with hydrogen peroxide. Ferumoxytol is widely used in preclinical and clinical studies because it has good safety and clearance profiles [3]. In terms of its usefulness in MPI, it has been proven to be useful in the quantitative imaging of transplanted stem cells. Ferumoxytol provides a longer blood half-life, more stable circulation kinetics, and a narrower size distribution compared to Resovist. However, it usually gives a lower MPI signal amplitude because of its different core size and magnetic relaxation properties. Thus, while Resovist is more efficient for strong, short-duration signal generation, ferumoxytol is better suited for applications requiring prolonged imaging windows and repeated measurements [70].

The findings indicate that LS-008 offers significant improvements in MPI applications, including enhanced image quality, clearer delineation of vascular structures, and longer circulation times. LS-008’s potential to significantly advance MPI technology and improve its diagnostic capabilities makes it a promising candidate for further development and clinical application.

Similar to how coating modifications affect MPI functionality, Zhou et al. [15] demonstrated that labeling MPI particles with specific materials could further enhance MPI functionality. In 2017, Zhou et al. [15] successfully targeted the pulmonary capillary bed by labeling SPION particles with Macro Aggregated Albumin (MAA). The MAA component, sized 10−90 μm, targets the lungs by being trapped in the first capillary bed encountered after IV injection, which has a 6-μm average lumen diameter. This labeling enabled the modification of MPI functionality to target specific tissues, facilitating more precise and safe diagnosis of tissue-specific conditions, such as life-threatening lung pathologies like PE [15].

In 2020, a newly developed multicore nanoparticle (MCP 3) was introduced by Mohtashamdolatshahi et al. as tracers for cardiovascular MPI. Mohtashamdolatshahi et al. also compared them to conventional tracers such as the previously mentioned Resovist and the MPI-tailored tracers (MNP LS-008) [16]. MCP 3 demonstrated superior spatial resolution, achieving at least a tenfold lower dosage than Resovist and at least a twofold lower dosage compared to the MPI-tailored MNP LS-008 in mice [71].

In 2020, a new multimodal imaging agent called 5-HT-Fe3O4-Cy7 nanoparticles (5HFeC NPs) demonstrated significant potential for the detection of vulnerable atherosclerotic plaques and monitoring active myeloperoxidase MPO (an inflammatory biomarker). Tong et al. employed fluorescence imaging (FLI) and magnetic particle imaging/computed tomographic angiography (MPI/CTA) in a mouse model with MPO implants to test how well these nanoparticles worked [72].

The 5-hydroxytryptamine (5-HT) component of the imaging agent can self-oligomerize or bind proteins when active MPO is present in inflamed tissues. This makes it even more likely to be used as an imaging biomarker for vulnerable plaques [73]. This property enhances the effectiveness of 5HFeC NPs in targeting and visualizing atherosclerotic plaques. Advanced stages of human atheromas are characterized by increased levels of MPO-expressing macrophages compared to early stage lesions, underscoring MPO’s significance as a tracer target for identifying vulnerable plaques [74]. These advancements in early plaque detection through MPI/CTA hold the potential to significantly reduce the incidence of arterial diseases and related complications, including plaque rupture and myocardial infarction, thereby preventing numerous cases of morbidity and mortality.

## 5. Expanding the Capabilities of MPI

A clinical imaging-capable MPI system must be sensitive enough to find even small amounts of SPIONs. There is a direct link between the Signal-to-Noise Ratio (SNR) of the measurement signal and spatial resolution. A highly sensitive MPI scanner is necessary to make MPI images with high resolution and improved signal-to-noise ratio [75].

Initial MPI systems had safety limitations, particularly a small FOV due to Specific Absorption Rate (SAR) and Peripheral Nerve Stimulation (PNS) constraints [76,77,78]. To address the small FOV issue, Vogel et al. [11] introduced the TWMPI as an alternative scanner concept in 2014. Further improvements in technology, as described above, have been focused on increasing the bore size enough to fit human extremities or a head, for example. Capable of scanning an entire mouse-sized volume simultaneously, TWMPI is ideal for rapid applications such as real-time measurements in small rodents, thanks to its swift data acquisition over a large FOV, encompassing an entire mouse-sized sample [11].

MPI’s main strengths are high temporal resolution and sensitivity. It is established that MPI can evaluate dynamic processes, including functional cardiac imaging, although the clinical applicability is still unknown. To make MPI even more sensitive, Graeser et al. constructed a very sensitive gradiometric receiver-coil unit with a noise-matching network for mouse imaging [61]. They conducted experiments involving the calibration of the coil with iron nanoparticle samples, system matrix acquisition, and image reconstruction using iterative algorithms. The setup detected 5 ng of iron in vitro in 2.14 s and an iron concentration of 156 μg/L, the lowest reported for an MPI scanner. In vivo, it captured the flow of an injected tracer through a mouse heart using a 512 ng bolus in 21.5 ms. The study concluded that MPI, with the advanced receiver coil, offers highly sensitive, rapid imaging suitable for real-time medical applications, particularly in cardiovascular diagnostics and interventions, providing a non-invasive, radiation-free alternative.

The primary obstacle in transitioning MPI from a preclinical setting to clinical use has been the absence of an imaging device with an adequately large bore size. Most systems documented in the existing literature feature bore diameters ranging from 3 cm to 12 cm, limiting their use to small animals like mice and rats. Previous efforts to construct a human-sized imager have revealed that achieving submillimeter resolution is technically challenging, requiring sophisticated and costly high-end hardware. Upscaling these systems for human application involves significant technical effort.

To solve this problem, Gräser et al. [62] created a human-sized MPI system in 2019 that was easy and quick to set up in clinical settings. The system had a bore size of 19 cm to 25 cm, which was perfect for an adult’s head and made for brain applications. It has low technical requirements while still addressing the clinical needs for monitoring cerebral diseases. This system can be put next to a bed in a stroke or intensive care unit, where space is often constrained by other life-support and monitoring devices. This system can be mounted bedside in stroke or intensive care units, enabling regular neurovascular status monitoring. Such bedside equipment can perhaps provide quantitative perfusion maps of the cerebral vasculature at higher temporal resolution. Their system’s compact size and lack of specific building requirements allow it to be set up as a mobile unit, facilitating diagnosis at the patient’s bedside [62].

Efforts are still underway to develop a human torso-sized MPI scanner to fit the human body. Vogel et al. [79] came up with the first portable interventional MPI (iMPI) system for real-time endovascular procedures in 2023. This system featured an innovative field generator that provides a substantial field of view and an open design tailored for integration with conventional X-ray angiography. The study demonstrated that real-time iMPI-guided Percutaneous Transluminal Angioplasty (PTA) was possible with a realistic, dynamic human-sized leg model. The findings suggest that the iMPI system offers a greatly promising and efficient solution for rapid, high-resolution imaging in clinical environments [79].

## 6. Challenges and Path Toward Clinical Translation

While MPI offers distinct advantages such as high sensitivity, absence of ionizing radiation, and linear quantitation of tracer concentration, several technical and translational barriers remain before routine human application can be realized. The most pressing engineering limitations involve SAR, PNS, and tissue heating, which restrict the drive-field amplitudes and frequencies that can be safely applied in human-scale systems [7,80]. These parameters directly affect achievable spatial and temporal resolution. Additionally, upscaling scanners from small-animal to human-sized bores introduces electromagnetic design challenges, energy consumption issues, and cost barriers [43]. From a materials perspective, most currently available SPIONs, such as Resovist and Ferumoxytol, were originally developed for MRI or therapeutic use and are not optimized for MPI. To support clinical translation, tracers must be synthesized under Good Manufacturing Practice (GMP) conditions, exhibit a narrow size distribution, have a long blood half-life, and be validated for biocompatibility. Regulatory approval will further require standardized toxicity studies, reproducibility data, and comparative safety profiles against existing MRI contrast agents [81].

Several strategies are under development to address these challenges. Recent innovations for scanners, such as adaptive drive-field modulation, low-SAR excitation sequences, and enhanced receiver coil configurations, aim to reduce PNS and heating while maintaining high gradient strength. Nanoparticle engineering for tracers focuses on biocompatible coatings, monodisperse cores, and multimodal hybrid tracers suitable for combined MPI-MRI systems [82]. Integration with AI-assisted image reconstruction and motion correction algorithms may further enhance temporal resolution and clinical usability [83,84].

The path toward clinical translation is expected to progress in defined phases. The first safety trials of MPI tracers on humans and the first certification of a pilot device for extremity and neurovascular scanners could happen in the next three to five years. Subsequently, larger multicenter studies evaluating diagnostic performance in cardiovascular and interventional applications were conducted. The anticipated regulatory trajectory will probably resemble that of MRI and PET systems, necessitating concurrent approval of both the equipment and the tracer by the FDA and the European Medicines Agency (EMA). Collaborative efforts among material scientists, imaging physicists, and clinicians will be essential to accelerate this transition from preclinical validation to bedside application. With these coordinated improvements, MPI has a good chance of becoming a quantitative, radiation-free, real-time imaging platform for cardiovascular diagnostics, perfusion mapping, and image-guided interventions in the next ten years. These considerations form the foundation for the future directions discussed in the following section.

## 7. Conclusions and Future Perspective

MPI has become a strong and radiation-free imaging technique and can directly and quantitatively demonstrate superparamagnetic tracers with extremely high temporal resolution [85]. In cardiovascular imaging, MPI facilitates real-time evaluation of myocardial perfusion, vascular patency, and device navigation, presenting functional data that is inaccessible through current modalities. The next step in MPI research should focus on clinical validation and integration, in addition to being technically feasible. To make sure that results can be reproduced and compared across platforms, it will be essential to establish standardized imaging protocols, quantitative reconstruction frameworks, and multicenter data harmonization. The real value of MPI compared to MRI, CT, and PET in specific cardiovascular and interventional contexts will be determined by collaborative clinical studies that focus on diagnostic accuracy, safety, and cost-effectiveness.

Another significant objective is to design clinical systems and integrate them into existing workflows. Compact scanner configurations, user-friendly interfaces, and hybrid platforms that merge MPI with MRI or CT could streamline adoption in clinical environments. Additionally, expanding the application to neurovascular, pulmonary, and oncological imaging will underscore MPI’s versatility beyond cardiology. In the field of research, ongoing improvements in data-driven modeling and AI-assisted interpretation may enhance image reconstruction, automate perfusion quantification, and make predictive analytics possible using dynamic tracer kinetics. Such computational tools will help translate MPI from high-precision laboratory imaging to real-time clinical decision support.

## Figures and Tables

**Table 1:** Quantitative Comparison Between MPI and Other Imaging Modalities.

Imaging Modality	Spatial Resolution	Temporal Resolution (Acquisition Time)	Quantitation	Hazard
CT	~0.5 mm	~1 s	Yes	Plain X-ray radiation
MRI	~1 mm	~1 s–1 h	No	Tissue Heating, Peripheral nerve stimulation, Magnetohydrodynamic effects, Interactions with implants, etc.
PET	~4 mm	~1 min	Yes	Ionizing γ Radiation
SPECT	~10 mm	~1 min	Yes	Ionizing γ Radiation
MPI	<1 mm	<0.1 s	Yes	Tissue heating, PNS *, Interactions with implants

* PNS: peripheral nerve stimulation.

**Table 2:** Summary of Preclinical Studies on Cardiac MPI.

Name	Year	Application	Experiment Type	Imaging Tracer	Conclusion	Ref.
Weizenecker et al.	2009	Features of a beating mouse heart revealed by the first in vivo 3D real-time MPI ^a^ images.	In Vivo	Resovist (SPION) ^b^ ferucarbotran)	A beating mouse heart can be imaged in vivo using MPI with high temporal and spatial resolutions.	[5]
Vogel et al.	2016	The viability of resolving the dynamics of a beating mouse heart in vivo utilizing a TWMPI ^c^ scanner.	In Vivo	Resovist (SPION ferucarbotran)	The dynamics of a beating mouse heart can be resolved with a high enough temporal resolution by a TWMPI scanner.	[11]
Kaul et al.	2017	Evaluating the performance of a new tracer, LS-008, for MPI against the standard Resovist to improve diagnostic imaging.	In Vitro and In Vivo	LS-008 & Resovist	LS-008 significantly improves MPI with better image quality, clearer vascular delineation, and longer circulation times, enhancing clinical imaging.	[49]

^a^ Magnetic particle imaging; ^b^ Superparamagnetic iron-oxide nanoparticles; ^c^ Traveling Wave Magnetic Particle Imaging.

**Table 3:** Key Studies on the Evolution and Improvement of MPI Tracers and Hardware Systems.

Name	Year	Application	Experiment Type	Imaging Tracer	Conclusion	Ref.
Haegele et al.	2014	Evaluation of the blood half-life of two different types of Resovist in MPI ^a^ to assess their applicability in cardiovascular MPI	In vivo	Resovist (SPION) ^b^ ferucarbotran)	Because the MPI signal from Resovist fades quickly, it is a suboptimal tracer for applications needing a longer presence of the MPI tracer in the blood pool.	[59]
Khandhar et al.	2015	Evaluation of the effects of PEG ^c^-based coatings on SPION stability and blood half-life.	In vivo	PEG-coated SPIONs PMAO-20kPEG	PEG coatings significantly improve colloidal stability, reduce protein adsorption, and prevent SPION aggregation, leading to a longer blood half-life of SPION.	[60]
Graeser et al.	2017	Enhancement of MPI sensitivity by developing a specialized receiver coil and integrating it into a commercial MPI scanner.	In vivo	MPI-tailored contrast agent	Integrating the advanced receiver coil into MPI enhances its sensitivity and precision as a rapid imaging modality suitable for real-time medical applications.	[61]
Graeser et al.	2019	Assessment of human-sized MPI’s sensitivity, spatial resolution, and stroke detection capability for brain applications	In vivo	Perimag	Human-sized MPI detects dynamic concentration changes and allows access to brain perfusion quantitatively in short intervals.	[62]

^a^ Magnetic particle imaging; ^b^ Superparamagnetic iron-oxide nanoparticles; ^c^ Polyethylene glycol.

## Data Availability

The paper contains all of the data.
